# Prevalence of multimorbidity in the Brazilian adult population according to socioeconomic and demographic characteristics

**DOI:** 10.1371/journal.pone.0174322

**Published:** 2017-04-06

**Authors:** Januse Nogueira de Carvalho, Ângelo Giuseppe Roncalli, Marianna de Camargo Cancela, Dyego Leandro Bezerra de Souza

**Affiliations:** 1 Collective Health Program, Federal University of Rio Grande do Norte, Natal, Rio Grande do Norte, Brasil; 2 Division of Population Research, Brazilian National Cancer Institute, Rio de Janeiro, Brasil; Heinrich-Heine-Universitat Dusseldorf Medizinische Fakultat, GERMANY

## Abstract

Knowledge on the occurrence of multimorbidity is important from the viewpoint of public policies, as this condition increases the consumption of medicines as well as the utilization and expenses of health services, affecting life quality of the population. The objective of this study was to estimate prevalence of self-reported multimorbidity in Brazilian adults (≥18 years old) according to socioeconomic and demographic characteristics. A descriptive study is presented herein, based on data from the National Health Survey, which was a household-based survey carried out in Brazil in 2013. Data on 60,202 adult participants over the age of 18 were included. Prevalences and its respective confidence intervals (95%) were estimated according to sex, age, education level, marital status, self-reported skin color, area of residence, occupation and federative units (states). Poisson regression models univariate and multivariate were used to evaluate the association between socioeconomic and demographic variables with multimorbidity. To observe the combinations of chronic conditions the most common groups in pairs, trios, quartets and quintets of chronic diseases were observed. The prevalence of multimorbidity was 23.6% and was higher among women, in individuals over 60 years of age, people with low educational levels, people living with partner, in urban areas and among unemployed persons. The states of the South and Southeast regions presented higher prevalence. The most common groups of chronic diseases were metabolic and musculoskeletal diseases. The results demonstrated high prevalence of multimorbidity in Brazil. The study also revealed that a considerable share of the economically active population presented two or more chronic diseases. Data of this research indicated that socioeconomic and demographic aspects must be considered during the planning of health services and development of prevention and treatment strategies for chronic diseases, and consequently, multimorbidity.

## Introduction

Developing countries currently experience an epidemiological transition, with declines in infectious diseases and constant increases in non-communicable diseases (NCD). NCD are among the main global causes of death, with examples such as cardiovascular diseases, neoplasms, mental disorders and diabetes[1]. In Brazil, extended longevity has increased the prevalence of these diseases, which can cause functional dependence and require repetitive hospitalizations[2]. Therefore, monitoring NCD must be an essential component during the planning, implementation and evaluation of health services, besides aiding in the elaboration of control and prevention measures[3].

A chronic disease is a disease that persists throughout a period above three months, and remains during a long life period, such as diabetes, hypertension, asthma and cancer [4,5]. Chronic diseases are characterized by a long lasting period, necessity of continuous medical treatment, severe impact on the affected individual, besides presenting high prevalence in older age groups [6].

Prevention, treatment and management of chronic diseases are complex, being one of the main challenges faced by health services worldwide[4]. Several studies have examined the independent effects of chronic diseases and the occurrence of comorbidities, defined as any additional co-existing ailment in a patient with a particular index disease [7]. In recent years, scientific evidence revealed that interactions between chronic diseases cause greater damage in life quality of people, more than could be expected from the individual effects of these conditions[8].

When two or more chronic diseases occur simultaneously in an individual, this is referred to as multimorbidity [9]. Knowledge on the occurrence of multimorbidity is especially important in primary attention, where medical doctors frequently assist patients with multiple, coexistent conditions [10]. Such knowledge is also important from the point of view of public policies, as these are conditions that increase the consumption of medicines as well as the utilization and expenses associated with health services.

Much has been learnt about the causes, prevention and treatment of NCDs over the past three decades, as important achievements have been made in reducing mortality in many countries; the evidence base for action is steadily mounting and global attention to the NCD epidemic is intensifying [11]. In this context, knowledge of multimorbidity becomes fundamental to subsidize health planning and conduct adequately combat chronic diseases. The objective of this study was to estimate the prevalence of multimorbidity in the Brazilian adult population according to socioeconomic and demographic characteristics.

## Methodology

A descriptive sectional study is presented herein. Data on the National Health Survey (NHS) were utilized, which was carried out in Brazil in 2013, as a representative cross-sectional survey of the Brazilian adult population (age ≥18 years), executed by the Health Ministry and the Brazilian Institute of Geography and Statistics (*In Portuguese*, IBGE) [12].

The sample was household-based, with samples stratified in three cluster stages: census sectors (primary units); households (second stage) and adult dwellers (third stage). The sample size considered the required precision level for estimations of some indicators in different disaggregation levels and population groups.

The minimum sample size of households per federative unit was 1,800 providing accurate estimates. The final weight was a product of the inverse of the selection probabilities at each stage of the sampling plan, including non-response correction procedures and adjustment calibrations for known population totals [12].

A total of 64,308 individuals aged 18 years or over were selected. The refusal rate was 2.7% (1,717 adults) and 3.7% of the selected adults (2,389) were not found. Only the interviews were kept in the study database, totaling 60,202 observations, corresponding to 93.6% of the sample. Further details about the NHS sample design and other methodological aspects can be found elsewhere[13].

The prevalence of multimorbidity was calculated by the number of interviewed adults that reported the diagnosis of two or more NCD in the NHS: systemic arterial hypertension; diabetes; hypercholesterolemia; heart issues (heart attack—infarction, angina or heart failure); cerebrovascular accident (CVA) or stroke; asthma; arthritis; vertebral spine issues (chronic pain in back or neck, sciatic pain, lumbago, issues with vertebrae or disks); work-related musculoskeletal disorders (WRMD); depression; mental issues such as schizophrenia, bipolar disorder, psychosis or obsessive-compulsive disorder (OCD); pulmonary diseases such as emphysema, chronic bronchitis or chronic obstructive pulmonary disease (COPD); cancer; and chronic kidney failure. These fourteen conditions were assessed through a question about have ever being diagnosed with the disease by a health professional. The specific question was, “Have you ever been told by a doctor that you have (disease name)? All of these chronic diseases utilized in the research were coded according to the standardized International Classification of Diseases and Related Health Problems (ICD-10). Fourteen morbidities were included, which were among the main diseases utilized to measure multimorbidity in a recent systematic review [14]. Multimorbidity was categorized for individuals presenting two, three, four and five chronic diseases. This questionnaire-based definition of multimorbidity has been widely used in several large internationally well-established epidemiological surveys, such as the Study on Global Ageing and Adult Health [15] and especially the WHO-World Health Surveys including Brazil [16]”.

Estimation of prevalences (%) and respective confidence intervals (95%) were carried out according to sex, age groups, education level, marital status, self-reported skin color, area of residence, occupation (employed or unemployed) and federative units (states). To observe the combinations of chronic conditions the most common clusters have been described in pairs, trios, quartets and quintets of chronic diseases, with their respective frequencies within each group. A univariate model (modified Poisson regression) were used to evaluate the association between socioeconomic and demographic variables with multimorbidity. The models statistically significant (p ≤ 0.05) by at least one stratum were entered into a multivariate. Modified Poisson regression (i.e., Poisson regression with a robust error variance) procedure is flexible and powerful as binomial regression, and the robust error estimate is used to deal with variance overestimation when Poisson regression is applied to binary data [17]. It was used measure of association prevalence ratio, obtained from the event coefficient (multimorbidity) in exposed and unexposed.

The program Stata version 14 was utilized for the statistical analysis, with the survey module for complex samples. The NHS was approved by the National Research Ethics Committee (NREC) of the National Health Council (NHC), Ministry of Health, approval number n°328.159 of June 26, 2013.

## Results

**Fig 1:**
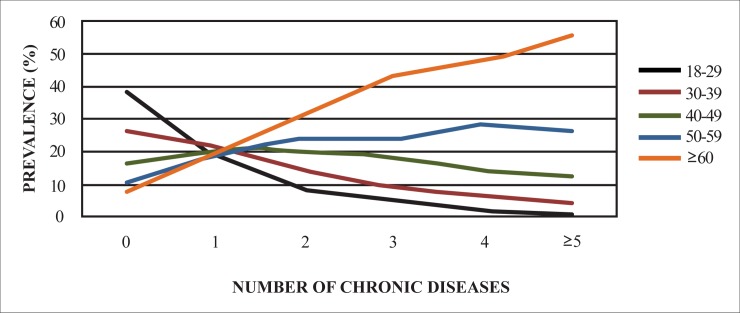
Prevalence (%) of chronic diseases by age in the adult Brazilian population—NHS, Brazil, 2013.

The prevalence of self-reported multimorbidity was 23.6%. The prevalence of interviewees that reported no diagnosis of chronic diseases was 52.0% and those that reported one chronic disease were 24.3%. From the number of adults within the sample that reported two or more chronic diseases, it was possible to estimate the number of cases for the Brazilian population over 18 years of age: 34,539,047 adults. It was observed that the number of chronic diseases increases with the age of the individuals. The observed prevalence of two or more chronic diseases was higher among individuals aged 60 years or older (51.2%) (Fig 1).The prevalence of multimorbidity increases with age for both men and women, however, women are more affected by multimorbidity in all age groups. The prevalence for two or more chronic diseases was higher among the individuals in the age group over 60 years (51.2%), people with low educational levels (37.2%), people living with partner (25.1%), people living in urban areas(24.1%) and among unemployed persons(33.6%). There was no statistically significant difference between skin colors (Table 1).

**Table 1:** Prevalence(%) of multimorbidity according to socioeconomic characteristics by sex. NHS, Brazil. 2013.

	Multimorbidity		
	Males %(CI_95%_^a^)	Females %(CI_95%_^a^)	Total %(CI_95%_^a^)
	n = 4,317	n = 9,152	n = 13,469
**Age (years)**			
18–29	4.2(3.5–5.1)	7.0(5.9–8.2)	5.6(4.9–6.4)
30–39	9.5(8.1–11.0)	14.8(13.4–16.3)	12.3(11.3–13.4)
40–49	16.0(14.3–17.8)	30.6(28.6–32.7)	23.9(22.6–25.3)
50–59	29.7(27.2–32.3)	42.6(40.2–45.0)	36.4(34.7–38.2)
≥60	43.4(40.7–46.2)	57.1(55.1–59.1)	51.1(49.5–52.8)
**Skin color**			
White	20.5(19.2–21.9)	30.2(28.8–31.6)	25.7(24.6–26.7)
Indigenous	18.1(9.3–32.3)	30.0(20.2–42.1)	25.1(17.9–34.0)
Black	17.5(14.6–20.9)	28.8(26.1–31.6)	23.5(21.5–25.6)
Yellow	22.4(14.3–33.3)	22.8(15.7–32.0)	22.6(16.9–29.6)
Brown	15.7(14.6–16.8)	26.3(25.1–27.6)	21.2(20.3–22.1)
**Education (years of study)**			
0–3	26.3(23.6–29.2)	46.4(43.3–49.4)	37.2(35.0–39.4)
4–7	22.4(20.9–24.0)	37.5(35.9–39.1)	30.1(28.9–31.3)
8–10	11.6(10.5–12.8)	19.1(17.9–20.5)	15.6(14.7–16.5)
≥11	17.6(15.5–19.8)	19.9(18.1–21.9)	18.9(17.4–20.4)
**Marital status (Living with a partner)**			
Yes	21.6(20.4–22.7)	28.6(27.3–29.8)	25.1(24.2–26.0)
No	12.2(11.1–13.3)	28.1(26.8–29.3)	21.1(20.2–22.1)
**Area of residence**			
Urban area	18.6(17.7–19.6)	28.9(27.9–29.9)	24.1(23.3–24.9)
Rural area	15.7(14.0–17.5)	24.7(22.7–26.8)	20.1(18.8–21.5)
**Employed**			
Yes	13.8(12.9–14.7)	21.9(20.8–23.1)	17.3(16.5–18.1)
No	31.1(29.1–33.2)	34.7(33.4–36.1)	33.6(32.5–34.8)
**Total**	18.2(17.3–19.0)	28.4(27.4–29.3)	23.6(22.9–24.3)

^a^ CI_95_%: confidence interval 95%

Among the individuals with multimorbidity, the chronic diseases with higher proportions were systemic arterial hypertension (63.0%), vertebral spine/back issues (49.4%), and hypercholesterolemia (43.0%) (Table 2).

**Table 2:** Proportion(%) of chronic diseases in the group of individuals with multimorbidity—NHS, Brazil. 2013.

Chronic disease	%
Systemic arterial hypertension	63.0
Issues with vertebral spine (chronic back or neck pain. sciatic pain. lumbago. issues with vertebrae or disks)	49.4
Hypercholesterolemia	43.0
Depression	25.0
Arthritis or rheumatism	23.1
Diabetes	21.6
Health issues (infarction, angina or heart failure)	15.6
Asthma or asthmatic bronchitis	11.7
Work-Related Musculoskeletal Disorder (WRMD)	7.3
Cancer	6.2
Lung issues, such as pulmonary emphysema, chronic bronchitis or chronic obstructive pulmonary disease (COPD)	6.0
Cerebrovascular accident (CVA) or stroke	5.7
Chronic kidney failure	4.8
Mental illnesses such as schizophrenia, bipolar disorder, psychosis or obsessive-compulsive disorder (OCD)	2.9

Regarding the number of chronic diseases within individuals that presented multimorbidity, a proportion of 52.8% individuals with two chronic diseases was observed; 25.8% presented three chronic diseases; 12.2% presented four chronic diseases, and 9.3% presented five or more chronic diseases. It was observed that, among the individuals with multimorbidity, the most common combination was hypertension and hypercholesterolemia (13.6%); among those that reported three diseases, the most common combination was hypertension, diabetes and hypercholesterolemia (9.0%); among those that reported four diseases, the most common combination was hypertension, diabetes, hypercholesterolemia and spine/back issues (6.7%); and among those that reported five diseases, the most common combination was hypertension, hypercholesterolemia, arthritis, back issues and depression (5.1%) (Table 3).

**Table 3:** Combination of chronic diseases among respondents with multimorbidity. NHS, Brazil, 2013.

Number of chronic diseases	Proportion(%)	Combination of chronic diseases
**2 diseases**	52.8	Hypertension+Hypercholesterolemia
		Hypertension+back issues
		Hypertension +diabetes
		Hypercholesterolemia + back issues
		Depression + back issues
**3 diseases**	25.8	Hypertension+diabetes+ Hypercholesterolemia
		Hypertension+ Hypercholesterolemia+ backissues
		Hypertension + back issues + arthritis
		Hypertension + backissues+depression
		Hypertension + diabetes + back issues
**4 diseases**	12.1	Hypertension+ diabetes + Hypercholesterolemia + back issues
		Hypertension + Hypercholesterolemia + arthritis + back issues
		Hypertension + Hypercholesterolemia +back issues +depression
		Hypertension + diabetes + Hypercholesterolemia + heart issues
		Hypertension + Hypercholesterolemia + heart issues + back issues
**5 + diseases**	9.3	Hypertension + Hypercholesterolemia + arthritis + back issues+ depression
		Hypertension +diabetes+ Hypercholesterolemia + arthritis + back issues
		Hypertension + Hypercholesterolemia+ diabetes + back issues +depression
		Hypertension + Hypercholesterolemia + diabetes + heart issues + back issues
		Hypertension + Hypercholesterolemia + heart issues+ arthritis + back issues

It was observed that people who have more multimorbidity are women, elderly, people with low education, living with a partner, living in urban areas and unemployed (Table 4).

**Table 4:** Univariate and multivariate analysis of the association between sociodemographic characteristics and multimorbidity. NHS, Brazil, 2013.

	Prevalence of multimorbidity(%)	PR (CI_95%_^a^)	PR(Adjusted)(CI_95%_^a^)
**Sex**			
Males	18.2	1	1
Females	28.4	1.55(1.47–1.64)**	1.44(1.37–1.52)**
**Age(years)**			
18–29	5.6	1	1
30–39	12.3	2.17(1.87–2.53) **	2.09(1.79–2.43)**
40–49	23.9	4.24(3.69–4.86) **	3.92(3.41–4.51)**
50–59	36.4	6.44(5.62–7.39) **	5.80(5.05–6.66)**
≥60	51.1	9.05(7.93–10.32) **	7.52(6.56–8.63)**
**Skin color**			
White	25.7	1	1
Indigenous	25.1	0.97(0.70–1.35)	0.94(0.86–1.03)
Black	23.5	0.91(0.83–1.00)	0.87(0.68–1.12)
Yellow	22.6	0.88(0.66–1.16)	0.93(0.89–0.98)
Brown	21.2	0.82(0.78–0.87) **	1.08(0.78–1.50)
**Education (years of study)**			
≥11	37.2	1	1
8–10	30.1	0.82(0.75–0.90) **	0.93(0.85–1.01)
4–7	15.6	1.59(1.45–1.73) **	1.10(1.01–1.18)*
0–3	18.9	1.96(1.78–2.16) **	1.04(0.94–1.14)
**Marital status (Living with a partner)**			
No	21.1	1	1
Yes	25.1	1.18(1.12–1.24) **	1.12(1.07–1.18)**
**Area of residence**			
Rural area	20.1	1	1
Urban area	24.1	1.20(1.11–1.29) **	1.24(1.15–1.34)**
**Employed**			
Yes	17.3	1	1
No	33.6	1.94(1.84–2.04) **	1.19(1.12–1.26)**

*p<0,01

**p<0,001

^a^ CI_95_%: confidence interval 95%;PR: prevalence ratio

Regarding the distribution per federative units, it was possible to observe higher prevalences in Southern states: Rio Grande do Sul (30.5%); Santa Catarina (29.3%); and Paraná (27.8%). The federative unit with lowest prevalence was Roraima (13.9%).(Table 5)

**Table 5:** Prevalence of multimorbidity among individuals over 18 years of age, per federative unit and federal district. PNS, Brasil, 2013.

Federative unit	Prevalence of multimorbidity%(CI_95%_^a^)
Rio Grande do Sul	30.5(28.0–33.2)
Santa Catarina	29.3(25.6–33.3)
Paraná	27.8(24.6–31.2)
Minas Gerais	26.0(23.5–28.6)
São Paulo	25.8(24.2–27.6)
Rio Grande do Norte	24.4(21.7–27.2)
Pernambuco	23.9(21.8–26.1)
Tocantins	23.9(21.1–27.0)
Rio de Janeiro	23.4(21.5–25.4)
Goiás	23.2(21.0–25.7)
Mato Grosso	22.6(19.6–25.9)
Mato Grosso do Sul	22.0(19.7–24.4)
Ceará	21.2(18.9–23.7)
Distrito Federal	20.2(18.1–22.6)
Alagoas	19.6(17.3–22.1)
Paraíba	19.6(17.4–22.1)
Espírito Santo	19.6(16.9–22.7)
Rondônia	19.5(16.3–23.2)
Sergipe	19.4(17.2–21.8)
Bahia	19.1(16.9–21.6)
Piauí	18.3(15.4–21.6)
Amapá	17.7(15.1–20.6)
Acre	16.7(14.5–19.2)
Maranhão	16.5(13.9–19.6)
Amazonas	16.4(14.5–18.5)
Pará	14.9(12.8–17.4)
Roraima	13.9(12.0–16.1)

^a^ CI_95_%: confidence interval 95%

## Discussion

Data of this research presented the prevalence estimations for multimorbidity, based on the NHS results for Brazilian adults that self-reported two or more chronic diseases. The multimorbidity reported by 23.6% of interviewed adults in Brazil is higher than what was observed in developed countries, such as Australia, where a 2006 study detected a 17.1% prevalence [4]. Despite the relevance of studying multimorbidity, there are still few investigations on prevalence in developing countries such as Brazil, nevertheless aimed directed to elderly or hospitalized patients [16,18].

Some factors associated with the presence or absence of multimorbidity were reported in scientific literature. Advanced age, female sex and low socioeconomic status were associated with higher probabilities of multimorbidity [19]. A statistically significant difference was observed herein for the female gender, when compared to the male gender, in all estimations. Besides, as the number of chronic diseases increased, prevalence also increased in women. The higher occurrence of multimorbidity in women has been reported by other researchers [19,20]. In Brazil, a study evaluated the presence of multimorbidity and associated factors in women aged 40 to 65 years with 11 or more years of school education. The reported morbid conditions evaluated was depression, hypertension, diabetes mellitus, urinary incontinence, and insomnia, using an anonymous self-report questionnaire completed by 377 women concluded that 39.3% reported two or more morbid conditions [20]. When multimorbidity occurred in women over the age of 50, it could be explained by higher vulnerability and higher exposure times to risk factors for chronic diseases [20]. Another explanation is that women are more sensitive to their health condition and therefore, tend to report more diseases [19]. Compared to women, men are more likely to die prematurely from non-communicable diseases which can lead to a higher prevalence among women [21].

The strong association between multimorbidity and age has been described in literature [22,23]. A statistically significant difference was revealed herein, regarding age, with a higher prevalence of multimorbidity in individuals of the age group ≥ 60 years. It was also observed that the number of chronic diseases and prevalence of multimorbidity increase according to age. Some multimorbidity prevalence studies focused on elderly population, especially because this is an important prognostic factor during hospitalizations and post-hospitalization survival[18]. A population-based study carried out in Canada indicated a multimorbidity prevalence between 40% and 50% in the population aged 65 years or more [24], which was similar to results found herein.

This study revealed that a significant contingent of the economically active Brazilian population presented multimorbidity, corresponding to 29.9% of the interviewees within the age group 40–59 years. A similar prevalence figure was observed in a study carried out in Scotland[22]. It is known that work-related issues can exert strong influence on health [25] Work-related musculoskeletal disorders are characterized as painful and harmful disorders caused by the use or excessive activity of some part of the musculoskeletal system, usually resulting from work-related physical activities. In Brazil, 2.4% reported a medical diagnosis of work-related musculoskeletal disorders, with women being the most affected when compared to men[26]. The high prevalence of multimorbidity in middle-aged persons can be a consequence of the greater proportion of this population in the work force, submitted to a higher load of chemical, physical, biological, mechanical and stressful risks besides work-related issues. This importance is paramount when considering the high demand for health services by this age group, besides the consequences that originate from chronic diseases, which can result in absenteeism. In the Brazilian public health system, primary care services are offered on weekdays, with no nocturnal hours or assistance on weekends, which can represent an access issue especially to the economically active population that cannot be assisted during mornings or afternoons.

A socioeconomic inequality, cited by literature as an associated factor to multimorbidity [27,28], is a strong characteristic of the Brazilian context, with extended consequences to health. It was possible to observe that the higher the number of chronic diseases, the higher the prevalence of people with low education levels. In this study the distribution of multimorbidity according to skin color followed the patterns of auto-declared racial and ethnic composition of Brazilian society [29], and there was no prevalence difference between groups.

It was possible to observe herein that there was higher multimorbidity prevalence concentration in federative units of the South region and lower prevalence of morbidity in the North region. The geographic differences verified for multimorbidity prevalence can be related to several factors such as different epidemiological characteristics, better or worse access to health services, cultural diversity and geographic characteristics deriving from the great territorial extension of Brazil, with irregularities in the distribution of population (more concentrated in the South and Southeast regions, and more sparse in the North region). The lower prevalence, especially in the North region, can be attributed to the fact that this region presents an overall younger population. Therefore, there are lower prevalences for chronic diseases and worse access to health services and specialized medical tests that could diagnose more easily the chronic conditions evaluated herein.

Regarding the most common chronic disease patterns in multimorbidity, a higher proportion of metabolic and musculoskeletal diseases were observed. The prevalence of diabetes and hypertension in Brazil is increasing, simultaneously with the prevalence of overweight[30]. Depression, other mental disorders and respiratory disorders appear next as similar disease clusters, also appearing in combination to the most prevalent chronic diseases in Brazil, especially when analyzing groups of three or more diseases. Similar combinations of diseases were obtained by research carried out in developed countries like United States, Germany and United Kingdom [31–33].

Care for people that present multimorbidity is a challenge for health systems. In Brazil, despite the existence of a universal health system that presents integral care as one of its principles, with a family-based primary care organized, it is still possible to observe a focused approach on medical specialties and more prevalent diseases such as hypertension and diabetes [34], than, cardiovascular disease and diabetes appear to be properly reported by individuals because of universal coverage by the Brazilian health system [35]. For the health care to individuals on the perspective of multimorbidity, multiprofessional teams should consider migrating from the assistance model with emphasis on healing procedures to an integral model focused on actions for health promotion and prevention. When caring for multimorbidity patients, interaction between the diseases must be considered in the sense of promoting better life quality and minimizing functional limitations, besides minimizing costs related to repetitive hospitalizations and other complications that result from multimorbidity. A generalist professional can be preferred, once multimorbidity patients are more susceptible to care fragmentation and medical errors [22].

### Limitations of the study

The study herein present utilized a database that represents the general population, presenting limitations, particularly because it addressed self-reported diagnosis data. Data loss could probably exist, especially in the regions with lower offer of health services, which implies that the findings underestimate the real prevalence of multimorbidity. In turn, research has demonstrated high agreement rates between self-report of health conditions and clinical evaluation of the presence or absence of non-communicable diseases, especially in individuals with higher education levels [20]. Another limitation in studies about multimorbidity concerns the lack of standardization of evaluated diseases that make it difficult to compare the results obtained.

## Conclusions

The results showed a high prevalence of people with two or more chronic diseases, indicating that multimorbidity is not an issue restricted to developed countries, also affecting countries undergoing epidemiological transitions, such as Brazil. The study also revealed a high prevalence of multimorbidity in the economically active population, suggesting that primary health care services should be organized in time periods to accommodate this representative contingent of the population. Socioeconomic and demographic aspects must be considered when planning health services and developing strategies for prevention and treatment of chronic diseases and, consequently, for multimorbidity. Policies oriented towards the reduction of inequities could favor the control of multimorbidity.

## Supporting information

S1 Table Prevalence(%) of multimorbidity according to socioeconomic characteristics by sex. NHS, Brazil. 2013.
(PDF)

S2 Table Proportion(%) of chronic diseases in the group of individuals with multimorbidity—NHS, Brazil.2013. (PDF)

S3 Table Univariate and multivariate analysis of the association between sociodemographic characteristics and multimorbidity. NHS, Brazil, 2013. (PDF)

S4 Table Univariate and multivariate analysis of the association between sociodemographic characteristics and multimorbidity. NHS, Brazil, 2013.
(PDF)

S5 Table Prevalence of multimorbidity among individuals over 18 years of age, per federative unit and federal district. PNS, Brasil, 2013.
(PDF)

S1 Fig Prevalence (%) of chronic diseases by age in the adult Brazilian population—NHS, Brazil, 2013. (EPS)
