# Supplementary material for: Prevalence of multimorbidity in the Brazilian adult population according to socioeconomic and demographic characteristics
Source: PLoS One. 2017 Apr 6;12(4):e0174322. doi: 10.1371/journal.pone.0174322 (PMC5383049; doi:10.1371/journal.pone.0174322)
Supplement: S1 Table — NHS, Brazil. 2013. (PDF) [file pone.0174322.s001.pdf]

Table 1 – Prevalence(%) of multimorbidity according to socioeconomic characteristics by sex. NHS, Brazil. 2013.

	Multimorbidity		
	**Males %($CI_{95\%}$[a])**	**Females %($CI_{95\%}$[a])**	**Total %($CI_{95\%}$[a])**
	**n=4,317**	**n=9,152**	**n=13,469**
**Age (years)**			
18-29	4.2(3.5-5.1)	7.0(5.9-8.2)	5.6(4.9-6.4)
30–39	9.5(8.1-11.0)	14.8(13.4-16.3)	12.3(11.3-13.4)
40–49	16.0(14.3-17.8)	30.6(28.6-32.7)	23.9(22.6-25.3)
50–59	29.7(27.2-32.3)	42.6(40.2-45.0)	36.4(34.7-38.2)
≥60	43.4(40.7-46.2)	57.1(55.1-59.1)	51.1(49.5-52.8)
**Skin color**			
White	20.5(19.2-21.9)	30.2(28.8-31.6)	25.7(24.6-26.7)
Indigenous	18.1(9.3-32.3)	30.0(20.2-42.1)	25.1(17.9-34.0)
Black	17.5(14.6-20.9)	28.8(26.1-31.6)	23.5(21.5-25.6)
Yellow	22.4(14.3-33.3)	22.8(15.7-32.0)	22.6(16.9-29.6)
Brown	15.7(14.6-16.8)	26.3(25.1-27.6)	21.2(20.3-22.1)
**Education (years of study)**			
0-3	26.3(23.6-29.2)	46.4(43.3-49.4)	37.2(35.0-39.4)
4–7	22.4(20.9-24.0)	37.5(35.9-39.1)	30.1(28.9-31.3)
8–10	11.6(10.5-12.8)	19.1(17.9-20.5)	15.6(14.7-16.5)
≥11	17.6(15.5-19.8)	19.9(18.1-21.9)	18.9(17.4-20.4)
**Marital status (Living with a partner)**			
Yes	21.6(20.4-22.7)	28.6(27.3-29.8)	25.1(24.2-26.0)
No	12.2(11.1-13.3)	28.1(26.8-29.3)	21.1(20.2-22.1)
**Area of residence**			
Urban area	18.6(17.7-19.6)	28.9(27.9-29.9)	24.1(23.3-24.9)
Rural area	15.7(14.0-17.5)	24.7(22.7-26.8)	20.1(18.8-21.5)
**Employed**			
Yes	13.8(12.9-14.7)	21.9(20.8-23.1)	17.3(16.5-18.1)
No	31.1(29.1-33.2)	34.7(33.4-36.1)	33.6(32.5-34.8)
**Total**	18.2(17.3-19.0)	28.4(27.4-29.3)	23.6(22.9-24.3)

[a] $CI_{95\%}$: confidence interval 95%
