# Supplementary material for: Prevalence of multimorbidity in the Brazilian adult population according to socioeconomic and demographic characteristics
Source: PLoS One. 2017 Apr 6;12(4):e0174322. doi: 10.1371/journal.pone.0174322 (PMC5383049; doi:10.1371/journal.pone.0174322)
Supplement: S2 Table — (PDF) [file pone.0174322.s002.pdf]

Table 2 – Proportion(%) of chronic diseases in the group of individuals with multimorbidity - NHS, Brazil. 2013.

**Chronic disease**	**%**
Systemic arterial hypertension	63.0
Issues with vertebral spine (chronic back or neck pain. sciatic pain. lumbago. issues with vertebrae or disks)	49.4
Hypercholesterolemia	43.0
Depression	25.0
Arthritis or rheumatism	23.1
Diabetes	21.6
Health issues (infarction, angina or heart failure)	15.6
Asthma or asthmatic bronchitis	11.7
Work-Related Musculoskeletal Disorder (WRMD)	7.3
Cancer	6.2
Lung issues, such as pulmonary emphysema, chronic bronchitis or chronic obstructive pulmonary disease (COPD)	6.0
Cerebrovascular accident (CVA) or stroke	5.7
Chronic kidney failure	4.8
Mental illnesses such as schizophrenia, bipolar disorder, psychosis or obsessive-compulsive disorder (OCD)	2.9
