# Supplementary material for: Prevalence of multimorbidity in the Brazilian adult population according to socioeconomic and demographic characteristics
Source: PLoS One. 2017 Apr 6;12(4):e0174322. doi: 10.1371/journal.pone.0174322 (PMC5383049; doi:10.1371/journal.pone.0174322)
Supplement: S3 Table — (PDF) [file pone.0174322.s003.pdf]

Table 3- Combination of chronic diseases among respondents with multimorbidity. NHS, Brazil, 2013.

**Number of chronic diseases**	**Proportion (%)**	**Combination of chronic diseases**
**2 diseases**	52.8	Hypertension+Hypercholesterolemia
		Hypertension+back issues
		Hypertension +diabetes
		Hypercholesterolemia + back issues
		Depression + back issues
**3 diseases**	25.8	Hypertension+diabetes+ Hypercholesterolemia
		Hypertension+ Hypercholesterolemia+ backissues
		Hypertension + back issues + arthritis
		Hypertension + backissues+depression
		Hypertension + diabetes + back issues
**4 diseases**	12.1	Hypertension+ diabetes + Hypercholesterolemia + back issues
		Hypertension + Hypercholesterolemia + arthritis + back issues
		Hypertension + Hypercholesterolemia +back issues +depression
		Hypertension + diabetes + Hypercholesterolemia + heart issues
		Hypertension + Hypercholesterolemia + heart issues + back issues
**5 + diseases**	9.3	Hypertension + Hypercholesterolemia + arthritis + back issues+ depression
		Hypertension +diabetes+ Hypercholesterolemia + arthritis + back issues
		Hypertension + Hypercholesterolemia+ diabetes + back issues +depression
		Hypertension + Hypercholesterolemia + diabetes + heart issues + back issues
		Hypertension + Hypercholesterolemia + heart issues+ arthritis + back issues
