# Supplementary material for: Prevalence of multimorbidity in the Brazilian adult population according to socioeconomic and demographic characteristics
Source: PLoS One. 2017 Apr 6;12(4):e0174322. doi: 10.1371/journal.pone.0174322 (PMC5383049; doi:10.1371/journal.pone.0174322)
Supplement: S4 Table — NHS, Brazil, 2013. (PDF) [file pone.0174322.s004.pdf]

Table 4- Univariate and multivariate analysis of the association between sociodemographic characteristics and multimorbidity. NHS, Brazil, 2013.

	**Prevalence of multimorbidity(%)**	**PR ($CI_{95\%}$[a])**	**PR(Adjusted)($CI_{95\%}$[a])**
**Sex**			
Males	18.2	1	1
Females	28.4	1.55(1.47-1.64)**	1.44(1.37-1.52)**
**Age(years)**			
18-29	5.6	1	1
30–39	12.3	2.17(1.87-2.53) **	2.09(1.79-2.43)**
40–49	23.9	4.24(3.69-4.86) **	3.92(3.41-4.51)**
50–59	36.4	6.44(5.62-7.39) **	5.80(5.05-6.66)**
≥60	51.1	9.05(7.93-10.32) **	7.52(6.56-8.63)**
**Skin color**			
White	25.7	1	1
Indigenous	25.1	0.97(0.70-1.35)	0.94(0.86-1.03)
Black	23.5	0.91(0.83-1.00)	0.87(0.68-1.12)
Yellow	22.6	0.88(0.66-1.16)	0.93(0.89-0.98)
Brown	21.2	0.82(0.78-0.87) **	1.08(0.78-1.50)
**Education (years of study)**			
≥11	37.2	1	1
8–10	30.1	0.82(0.75-0.90) **	0.93(0.85-1.01)
4-7	15.6	1.59(1.45-1.73) **	1.10(1.01-1.18)*
0-3	18.9	1.96(1.78-2.16) **	1.04(0.94-1.14)
**Marital status (Living with a partner)**			
No	21.1	1	1
Yes	25.1	1.18(1.12-1.24) **	1.12(1.07-1.18)**
**Area of residence**			
Rural area	20.1	1	1
Urban area	24.1	1.20(1.11-1.29) **	1.24(1.15-1.34)**
**Employed**			
Yes	17.3	1	1
No	33.6	1.94(1.84-2.04) **	1.19(1.12-1.26)**

*p<0,01 **p<0,001

[a] $CI_{95\%}$: confidence interval 95%;PR: prevalence ratio
