# Supplementary material for: Prevalence of multimorbidity in the Brazilian adult population according to socioeconomic and demographic characteristics
Source: PLoS One. 2017 Apr 6;12(4):e0174322. doi: 10.1371/journal.pone.0174322 (PMC5383049; doi:10.1371/journal.pone.0174322)
Supplement: S5 Table — PNS, Brasil, 2013. (PDF) [file pone.0174322.s005.pdf]

Table 5- Prevalence of multimorbidity among individuals over 18 years of age, per federative unit and federal district. PNS, Brasil, 2013.

**Federative unit**	**Prevalence of multimorbidity%($CI_{95\%}$[a])**
Rio Grande do Sul	30.5(28.0-33.2)
Santa Catarina	29.3(25.6-33.3)
Paraná	27.8(24.6-31.2)
Minas Gerais	26.0(23.5-28.6)
São Paulo	25.8(24.2-27.6)
Rio Grande do Norte	24.4(21.7-27.2)
Pernambuco	23.9(21.8-26.1)
Tocantins	23.9(21.1-27.0)
Rio de Janeiro	23.4(21.5-25.4)
Goiás	23.2(21.0-25.7)
Mato Grosso	22.6(19.6-25.9)
Mato Grosso do Sul	22.0(19.7-24.4)
Ceará	21.2(18.9-23.7)
Distrito Federal	20.2(18.1-22.6)
Alagoas	19.6(17.3-22.1)
Paraíba	19.6(17.4-22.1)
Espírito Santo	19.6(16.9-22.7)
Rondônia	19.5(16.3-23.2)
Sergipe	19.4(17.2-21.8)
Bahia	19.1(16.9-21.6)
Piauí	18.3(15.4-21.6)
Amapá	17.7(15.1-20.6)
Acre	16.7(14.5-19.2)
Maranhão	16.5(13.9-19.6)
Amazonas	16.4(14.5-18.5)
Pará	14.9(12.8-17.4)
Roraima	13.9(12.0-16.1)

[a] $CI_{95\%}$: confidence interval 95%
